# Generation of a Hutchinson–Gilford progeria syndrome monkey model by base editing

**DOI:** 10.1007/s13238-020-00740-8

**Published:** 2020-07-29

**Authors:** Fang Wang, Weiqi Zhang, Qiaoyan Yang, Yu Kang, Yanling Fan, Jingkuan Wei, Zunpeng Liu, Shaoxing Dai, Hao Li, Zifan Li, Lizhu Xu, Chu Chu, Jing Qu, Chenyang Si, Weizhi Ji, Guang-Hui Liu, Chengzu Long, Yuyu Niu

**Affiliations:** 1 Yunnan Key Laboratory of Primate Biomedical Research, Institute of Primate Translational Medicine, Kunming University of Science and Technology, Kunming, 650500 China; 2 Faculty of Life Science and Technology, Kunming University of Science and Technology, Kunming, 650500 China; 3 Institute for Stem Cell and Regeneration, Chinese Academy of Science, Beijing, 100101 China; 4 CAS Key Laboratory of Genomic and Precision Medicine, Beijing Institute of Genomics, Chinese Academy of Sciences, Beijing, 100101 China; 5 China National Center for Bioinformation, Beijing, 100101 China; 6 University of Chinese Academy of Sciences, Beijing, 100049 China; 7 The Leon H Charney Division of Cardiology, New York University School of Medicine, New York, NY 10016 USA; 8 State Key Laboratory of Stem Cell and Reproductive Biology, Institute of Zoology, Chinese Academy of Sciences, Beijing, 100101 China; 9 State Key Laboratory of Membrane Biology, Institute of Zoology, Chinese Academy of Sciences, Beijing, 100101 China; 10 Advanced Innovation Center for Human Brain Protection, and National Clinical Research Center for Geriatric Disorders, Xuanwu Hospital Capital Medical University, Beijing, 100053 China; 11 Department of Neuroscience and Physiology, New York University School of Medicine, New York, NY 10016 USA; 12 Department of Neurology, New York University School of Medicine, New York, NY 10016 USA

**Keywords:** base editing, non-human primate, HGPS

## Abstract

Many human genetic diseases, including Hutchinson-Gilford progeria syndrome (HGPS), are caused by single point mutations. HGPS is a rare disorder that causes premature aging and is usually caused by a *de novo* point mutation in the *LMNA* gene. Base editors (BEs) composed of a cytidine deaminase fused to CRISPR/Cas9 nickase are highly efficient at inducing C to T base conversions in a programmable manner and can be used to generate animal disease models with single amino-acid substitutions. Here, we generated the first HGPS monkey model by delivering a BE mRNA and guide RNA (gRNA) targeting the *LMNA* gene via microinjection into monkey zygotes. Five out of six newborn monkeys carried the mutation specifically at the target site. HGPS monkeys expressed the toxic form of lamin A, progerin, and recapitulated the typical HGPS phenotypes including growth retardation, bone alterations, and vascular abnormalities. Thus, this monkey model genetically and clinically mimics HGPS in humans, demonstrating that the BE system can efficiently and accurately generate patient-specific disease models in non-human primates.

**Electronic supplementary material:**

The online version of this article (10.1007/s13238-020-00740-8) contains supplementary material, which is available to authorized users.

## Introduction

The vast majority of human genetic diseases are caused by single-nucleotide substitutions or point mutations (Landrum et al., 2016). These include the dozens of diseases collectively termed “laminopathies”, which are caused by a variety of mutations in the genes encoding the nuclear lamina proteins (Liu et al., 2011b). A premature aging human disorder, HGPS, is caused by a mutant *LMNA* gene (Capell and Collins, 2006; Liu et al., 2011a; Kubben et al., 2016). Approximately 90% of HGPS cases are caused by a *de novo* mutation (1824 C>T, Gly608Gly) in *LMNA*, which activates a cryptic splice donor site, resulting in an mRNA that lacks 150 nucleotides. The resultant mRNA is subsequently translated into a truncated prelamin A without the *ZMPSTE24* cleavage site, generating a toxic protein called “progerin”. The accumulation of progerin leads to pathologies associated with premature aging including growth impairment, dermal and bone abnormalities, lipodystrophy, and progressive atherosclerosis, all of which lead to a shortened lifespan, frequently via myocardial infarction.

Genetically engineered animal models, specifically non-human primates, are a valuable tool used to study human diseases and develop preclinical therapeutic strategies (Chan, 2013). Several recent studies have shown that the clustered regularly interspaced short palindromic repeats (CRISPR)/Cas9 system could be used to generate gene-knockout or gene-knockin monkeys (Kang et al., 2019). However, due to the low frequency of homologous recombination (HR) in the presence of a donor DNA template, the precise genome editing strategy, especially a single base-pair modification, remains a challenge. Recent improvements in base editing techniques have facilitated the direct and permanent conversion of a base pair in a programmable manner without introducing a double-strand break, which can lead to off-target mutagenesis and/or reduce cell viability due to DNA repair activation (Koblan et al., 2018b; Pickar-Oliver and Gersbach, 2019). Cytidine BEs enable single-nucleotide C- to -T conversions, which can install or correct pathogenic SNPs in much higher editing frequencies in a variety of mammalian cell types (Komor et al., 2016; Kim et al., 2017; Liang et al., 2017; Zhou et al., 2017; Koblan et al., 2018b; Liu et al., 2018a). The recently optimized BE, BE4max, exhibited improvements in gene expression and nuclear localization as well as highly increased editing efficiency (Koblan et al., 2018a). Here, we employed BE4max to explore the possibility of generating a *LMNA* (1824 C>T, Gly608Gly) mutational *Macaca fascicularis* (cynomolgus monkey) model for HGPS.

## Results

**Figure 1:**
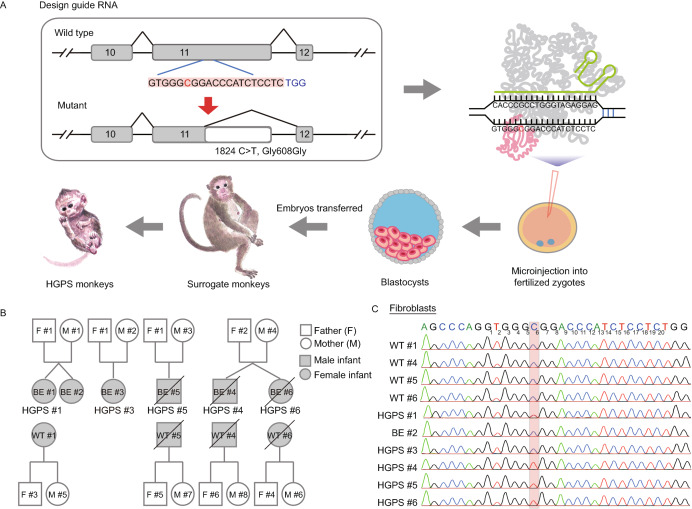

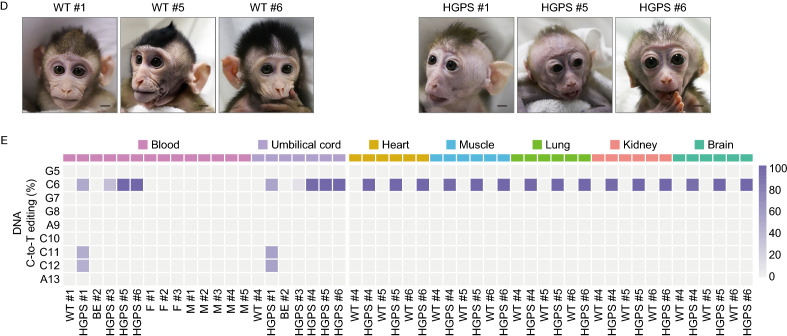
**Generation of HGPS monkeys.** (A) The schematic showed the process of generating HGPS monkeys. (B) Family tree of all monkeys used in this study. Black slash indicated that the monkey was dead (HGPS #4 died before the caesarean operation. HGPS #5 and HGPS #6 died when they were five months old.). (C) Sequencing of the sgRNA-targeted regions in the *LMNA* gene of fibroblasts from WT monkeys and HGPS monkeys. (D) Photographs of WT monkeys and HGPS monkeys when they were 3-months old. Scale bar, 0.83 cm. (E) Heat maps showed on-target editing efficiencies in various tissues of each monkey

A single guide RNA (sgRNA) was designed to introduce the *LMNA* (1824 C>T, Gly608Gly) mutation and was co-injected into 86 monkey zygotes along with BE4max mRNA. Then, 41 well-developed embryos injected with sgRNA and BE4max mRNA that displayed normal morphology were transferred into 11 surrogate mothers (Figs. 1A and S1A). Six surrogates were successfully impregnated. Five of the six completed the pregnancy cycle (~150 days) and successfully birthed one infant (referred as to BE #1, 2, 3, 5, and 6, respectively, Fig. 1B) via caesarean delivery. One male infant (BE #4) died before the caesarean operation (~150 days). Two monkeys (BE #5 and BE #6, female and male, respectively) died when they were five months old (Figs. 1B and Fig. S1A; Table S1).

PCR and Sanger sequencing were performed to dissect the flanking sequences of the target loci of the sgRNA. Genotyping of fibroblasts and peripheral blood isolated from the genetically engineered monkeys showed that five out of six monkeys (BE #1, BE #3, BE #4, BE #5, and BE #6) carried the expected C to T mutation at position 6, the target locus, and were subsequently renamed HGPS #1, HGPS #3, HGPS #4, HGPS #5, and HGPS #6 (Figs. 1C, 1D, S1C; Table S2). Notably, three (HGPS #4, #5, and #6) out of the six monkeys were homozygous for the expected nonsense mutation at the target site, and the single C-to-T conversion successfully generated cryptic splice sites (Figs. 1C and S1C). HGPS #1 and HGPS #3 were mosaic mutants (Figs. 1C, S1B and S1C).

To further evaluate the on-targeting efficiency of the *LMNA* locus, we performed whole-genome DNA sequencing (WGS) for six tissues isolated from HGPS and wild type (WT) monkeys, respectively. Chromosomal variation analysis did not reveal any genomic instability (Fig. S2). The editing efficiencies of the 1824 C>T (position 6) conversion were up to 100% in all three homozygous monkeys (HGPS #4, #5, and #6), and did not affect other nucleotides flanking the canonical base editing window (Figs. 1E, S1D, S1E, and Table S2). HGPS #1 harbored an editing frequency of around 50% at the target site (position 6) with two additional endogenous sites containing cytidines at positions 11 and 12 that also harbored the same C>T mutation in a heterozygous manner (Figs. 1E, S1D, S1E, and Table S2). Mutant monkey model (HGPS #3) harbored a mutation frequency of 11%–35% at the *LMNA* locus (Figs. 1E, S1D, S1E, and Table S2). To evaluate whether base editing in monkey zygotes results in genetic mosaicism after birth, we isolated five additional tissue types from the three homozygous HGPS monkeys (HGPS #4, #5, and #6) immediately after individual death, and subjected them to WGS. All tissues of the three monkeys were 100% edited, and we detected no WT version of *LMNA* by sequencing (Figs. 1E, S1F–H, and Table S2).

To characterize the potential genome-wide effects of base editing in newborn monkeys, we performed both gRNA-dependent and deaminase-dependent off-target analysis for BE4max. First, the potential genome-wide off-target sites for sgRNA were predicted by Cas-OFFinder (Bae et al., 2014) and analyzed by searching the WGS dataset for promiscuous editing. These sites in HGPS monkeys did not reveal any notable sequence alterations, in comparison to the WT (Table S3). For the deaminase-dependent off-target analysis, WGS analysis was performed in both HGPS and WT monkeys to identify fetus-specific *de novo* single nucleotide variations (SNVs) and indels. The genome-wide number and proportion of SNVs in both HGPS and WT offspring were extracted by referencing the SNVs of corresponding parents. Comparison of the WT and HGPS monkeys indicated no difference in the number or proportion of *de novo* SNVs at the genome-wide level (Fig. S3). Given that this comprehensive WGS analysis failed to detect any gRNA-independent random conversions, BE4max appears to be an effective base editor in the cynomolgus monkey. A previous study reported that the cytosine base editor 3 (BE3) induced *de novo* SNVs in mouse embryos via genome-wide off-target analysis by two-cell embryo Injection (GOTI) (Zuo et al., 2019). However, this method is difficult to perform in non-human primates, especially throughout the embryonic stages. In a recent attempt to study human embryos, the off-target effects of BE3 were analyzed only within the 8-cell stage embryos by GOTI (Zhang et al., 2019a). Therefore, to accurately evaluate both Cas9-dependent and deaminase-dependent off-target mutations by base editing in newborn monkeys, further WGS strategies, which are technically similar to the GOTI method, will be needed.

**Figure 2:**
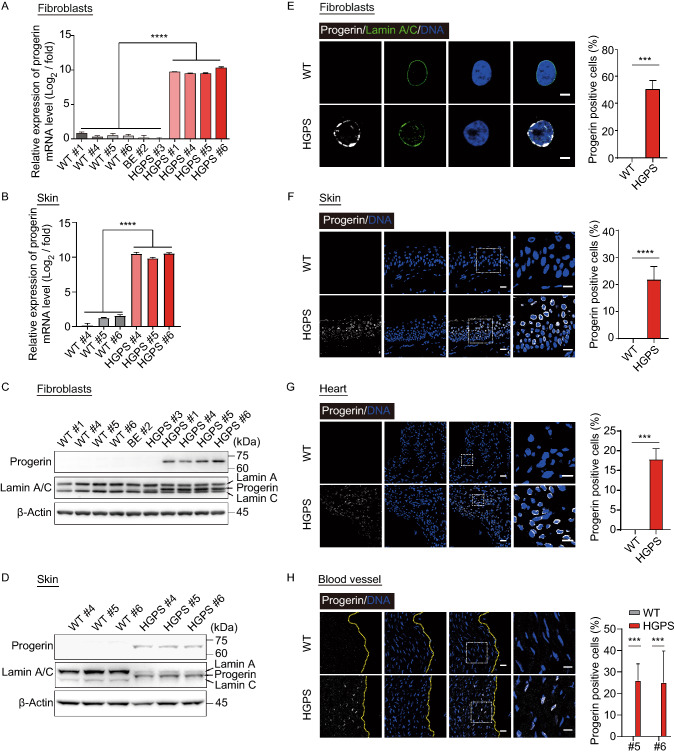
**Expression of progerin in fibroblasts and skin of HGPS monkeys.** (A and B) Quantitative analysis of progerin mRNA expression in the fibroblasts (A) and skin (B) of WT and HGPS by qPCR. The data from the HGPS monkeys were normalized to the corresponding data obtained from the WT monkeys. Data shown as mean ± SD, *n* = 4 wells per condition, ****P < 0.0001 (*t*-test). (C and D) Western blots showed the expression of progerin in the fibroblasts (C) and skin (D) of HGPS monkeys. For uncropped gels, refer to Source Data. (E–H) Immunofluorescence staining showed the expression of the progerin in the fibroblasts (E), skin (F), heart (G), and aorta (H) of HGPS monkeys. Right panels: the percentages of progerin positive cells. Scale bar, 25 μm, (zoom: 10 μm). Data are mean ± SD. *n* = 3 monkeys (WT #4, #5, #6 versus HGPS #4, #5, #6) for (E–G) and *n* = 2 monkeys (WT #5, #6 versus HGPS #5, #6) for (H). ***P < 0.001, ****P < 0.0001 (*t*-test for E, F, G and one-way ANOVA for H)

Next, we examined whether BE4max-mediated 1824 C>T editing in *LMNA* results in the production of progerin in monkeys. Quantitative RT-PCR (qRT-PCR) showed that progerin-specific mRNA was highly expressed in the fibroblasts, skin and the other nine tissues, such as heart and muscle, sampled from homozygous (HGPS #4, #5, and #6) and heterozygous HGPS monkeys (HGPS #1), but not in those from low efficiently edited or non-edited monkeys (HGPS #3 and BE #2), and their age and gender-matched WT counterparts (Figs. 2A, 2B, and S4A). Similar to the qRT-PCR results, Western blot and immunofluorescence analyses both demonstrated the presence of progerin in HGPS fibroblasts and tissues but not in WT tissues (Fig. 2C–H). Progerin was expressed in a tissue-specific manner, with high levels in the skin, heart, and blood vessels, which are known tissues affected by HGPS (Figs. 2D–H and S4B–F) (Aktas et al., 2013; Ullrich and Gordon, 2015). Progerin and Lamin A/C were hardly detected in the brain (Fig. S4D), which is consistent with the neuronal *LMNA* silencing and absence of cognitive defects in HGPS patients (Jung et al., 2012). Although HGPS #3 exhibited a mutation frequency of roughly 11%–35%, progerin was nearly undetectable in the fibroblasts (Fig. 2A and 2C). These results demonstrate that the engineered heterozygous and homogenous *LMNA* 1824 C>T monkeys, like HGPS patients, express pathogenic levels of progerin across various tissues. Interestingly, homozygous mice with the Gly609Gly mutation live for roughly 3 months and recapitulate the premature aging of HGPS (Osorio et al., 2011). Homozygous HGPS human stem cells also exhibit the accelerated cellular senescence, compared to their WT counterparts (Wu et al., 2018).

**Figure 3:**
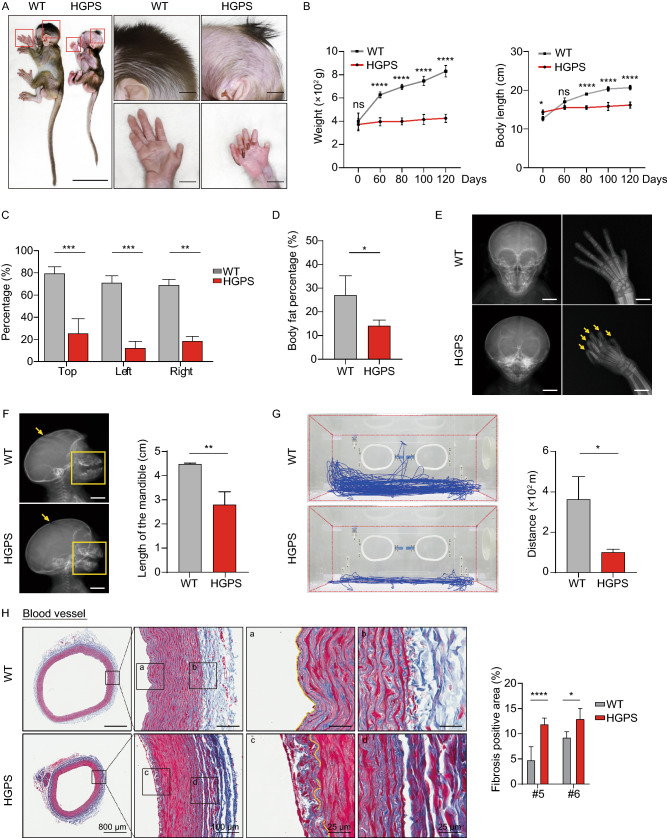
**HGPS monkeys exhibited clinical features of HGPS children.** (A) Representative photographs showing the appearance of WT monkey (WT #6) and HGPS monkey (HGPS #6) at 87 days of age. The typical phenotypes of growth retardation, bone abnormalities, and hair loss were overserved in HGPS monkeys. Scale bar: left panel, 9 cm; right panels, 4.5 cm. (B) Body weight and length of WT and HGPS monkeys after birth. Data are displayed as mean ± SD, *n* = 3 (WT #1, #5, #6 versus HGPS #1, #5, #6), ^ns^*P* > 0.5, *P < 0.5, ****P < 0.0001 (two-way ANOVA). (C) Quantitative analysis of hair loss in WT monkeys and HGPS monkeys at 100 days of age. The percentage of the area with hair was calculated in the top, left, and right side of the monkey head. Data were presented as mean ± SD, *n* = 3 (WT #1, #5, #6 versus HGPS #1, #5, #6), **P < 0.01, ****P < 0.0001 (one-way ANOVA). (D) Body fat percentage of HGPS monkeys and WT monkeys measured by dual-energy X-ray absorptiometry (DXA). Data were presented as mean ± SD, *n* = 3 or 5 (5 WT monkeys versus HGPS #1, #5, #6), *P < 0.5 (two-tailed Student’s *t*-test). (E) The radiographs of the skull anteroposterior of a WT monkey (WT #6) and a HGPS monkey (HGPS #6). Showing disproportionate large calvarium and contractures finger bone (indicated by yellow arrows) in the HGPS monkey. (F) The smaller mandible (yellow box) and open anterior fontanel (yellow arrow) of the HGPS monkey revealed by skull radiography. Data (right) were presented as mean ± SD, *n* = 3 (WT #1, #5, #6 versus HGPS #1, #5, #6), **P < 0.01 (*t-*test). (G) Decreased range of motion in HGPS monkeys determined by the motion tracking. Data are presented as mean ± SD, *n* = 3 (WT #1, #5, #6 versus HGPS #1, #5, #6), *P < 0.05 (*t*-test). (H) Masson’s trichrome staining of the aorta showing early features of atherosclerosis (c) and vascular fibrosis (d) in HGPS monkeys. Scale bar, 800 μm, 100 μm, 25 μm, 25 μm. *n* = 2 slices per monkeys (WT #5, #6 versus HGPS #5, #6). Data are mean ± SD, ****P < 0.0001 (one-way ANOVA)

We subsequently investigated whether the characteristic clinical phenotypes of HGPS patients were recapitulated in HGPS monkeys. Highly similar to HGPS human infants (Korf, 2008; Merideth et al., 2008), which appear normal at birth but grow into so-called “wizened dwarves” (Capell and Collins, 2006; Merideth et al., 2008; Ullrich and Gordon, 2015), the three liveborn HGPS monkeys (HGPS #1, #5, #6) had a normal stature at birth, but failed to thrive as WT monkeys (Fig. 3A and 3B). WT monkeys showed an average weight increase of 119 g/month in the first six months of life, whereas HGPS monkeys gained only 15 g/month with a normal head circumference (Figs. 3A, 3B, and S5A). Although HGPS monkeys were born with normal hair covering, excessive hair loss began in the temporal area one month after birth (Fig. 3C). HGPS monkeys also suffered from loss of subcutaneous fat (Fig. 3D). At two months post-birth, all three liveborn HGPS monkeys developed the characteristic craniofacial deformations, including a large bald skull, prominent forehead, protuberant eyes, and a hypoplastic mandible (Fig. 3E and 3F), which mirrors characteristics seen in HGPS patients (Capell and Collins, 2006; Merideth et al., 2008; Ullrich and Gordon, 2015).

HGPS monkeys were unable to extend their fingers (Figs. 3A, 3E, and S5), indicating joint contractures similar to those present in their human counterparts (Ozonoff and Clemett, 1967). Radiographs showed skeletal system aberrations, including overcrowded and small sharpened teeth and a short chin (Fig. 3E and 3F). Although HGPS monkeys began sitting, standing, and walking, bone damage greatly affected their motor abilities, characterized by a notable decrease in distance traveled in the infant incubators (Figs. 3G, S5C and S5D). Serum analysis revealed levels of blood glucose, cholesterol, triglyceride, liver and kidney function (such as alanine aminotransferase [ALT] and aspartate aminotransferase [AST]), and growth hormones, all of which were within the normal range (Fig. S5E and Table S4). All of the HGPS mutant phenotypes appeared to replicate the clinical manifestations of HGPS in humans (Fig. S5F) (Ozonoff and Clemett, 1967; Khalifa, 1989; Monu et al., 1990; Erdem et al., 1994; Stehbens et al., 1999; Gordon et al., 2005; Hennekam, 2006; Merideth et al., 2008; Rastogi and Chander Mohan, 2008; Doubaj et al., 2011; Gordon et al., 2011; Ullrich et al., 2012; Silvera et al., 2013; Chu et al., 2015; Ullrich and Gordon, 2015; Rivera-Torres et al., 2016; Prakash et al., 2018; Xu and Jin, 2019).

HGPS children suffer from global atherosclerosis and vascular complications (Xu and Jin, 2019). To dissect the early onset of disease-associated changes at the tissue level, a histopathological examination was performed on aortic biopsies of the deceased HGPS monkeys at five months old, which is equivalent to the human age of 1.5 years. Identical aortic anatomy was isolated from HGPS monkeys and their age-matched WT counterparts. An increase in vascular wall fibrosis was observed in the HGPS monkeys, which is associated with increased collagen formation and vessel stiffness during normal and pathological aging (Fig. 3H) (Selvin et al., 2010). Besides, the growing endothelium lesion began to encroach on the arterial lumen of HGPS monkeys, which is made evident by the appearance of intimal hyperplasia (Fig. 3H). These histological studies demonstrate that the HGPS monkey model captures the early events of atherosclerosis, which may help identify new biomarkers and preventative interventions to extend the lifespan of HGPS patients, and other individuals susceptible to atherosclerosis.

**Figure 4:**
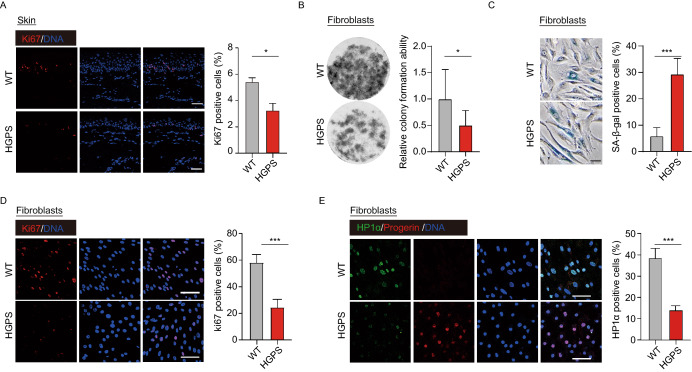
***LMNA***^**G608G**^ **resulted the decrease of cell proliferation ability.** (A) Immunofluorescence staining of Ki67 demonstrated reduced proliferation of HGPS monkeys’ skin cells. Scale bar, 25 μm. *n* = 4 (WT #1, #4, #5, #6 versus HGPS #1, #4, #5, #6). Data are mean ± sd., *P < 0.05 (*t*-test). (B) The clonal images showed reduced expansion ability of the HGPS monkeys’ fibroblasts (*n* = 4 monkeys WT #1, #4, #5, #6 versus HGPS #1, #4, #5, #6). Data are mean ± SD, *P < 0.05 (*t*-test). (C) Immunofluorescence staining of SA-β-gal demonstrated increased senescence of HGPS monkeys’ fibroblasts. Scale bar, 50 μm. *n* = 4 (WT #1, #4, #5, #6 versus HGPS #1, #4, #5, #6). Data are mean ± SD, ***P < 0.001 (*t*-test). (D) Immunofluorescence staining of Ki67 demonstrated a decrease in proliferation of HGPS monkeys’ fibroblasts. Scale bar, 75 μm. *n* = 4 (WT #1, #4, #5, #6 versus HGPS #1, #4, #5, #6). Data are mean ± SD, ***P < 0.001 (*t*-test). (E) HP1α’s immunofluorescence staining of fibroblasts demonstrated heterochromatin loss in HGPS monkeys. Scale bar, 75 μm. *n* = 4 (WT #1, #4, #5, #6 versus HGPS #1, #4, #5, #6). Data are mean ± SD, ***P < 0.001 (*t*-test)

**Figure 5:**
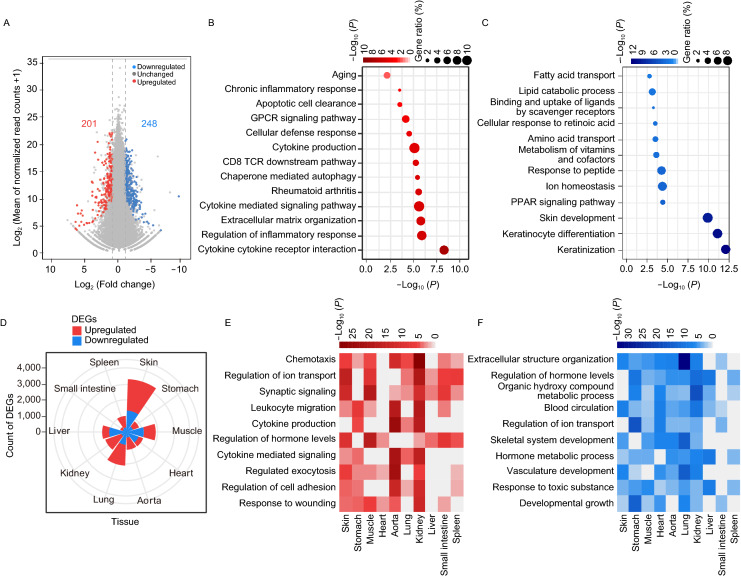
**Transcriptome features in HGPS monkeys.** (A) Scatter plot showed the DEGs between the skin samples of WT (WT#1, WT#5 and WT#6) and HGPS (HGPS #1, HGPS #5 and HGPS #6) monkeys. The number in red showed the count of upregulated DEGs [log_2_ (Fold change) > 1, adjusted-*P* < 0.05]; the number in blue shows the count of downregulated DEGs [log_2_ (Fold change) < −1, adjusted-*P* < 0.05]. (B and C) Dot plot showed the enriched GO-terms or pathways for upregulated (B) and downregulated (C) genes in skin samples of HGPS (HGPS #1, #5 and #6) compared to WT (WT #1, #5 and #6) monkeys. The color key from white to red (B) and white to blue (C) indicates low to high enrichment level [-log_10_ (*P*-value)] for each GO-term or pathway. The circle size indicates to the ratio of genes enriched in the GO-term or pathway. (D) Wind-rose plot showed the numbers of DEGs between WT (WT #5 and WT #6) and HGPS (HGPS #5 and HGPS #6) monkeys in various tissues. Red represents the count of upregulated genes and blue represents the count of downregulated genes between WT monkeys and HGPS monkeys. (E and F) Heat maps showed the enriched GO-terms or pathways for upregulated (E) and downregulated (F) in tissues of the HGPS #5 and HGPS #6 monkeys compared with the matched WT #5 and WT #6 monkeys. The color keys from white to red (E) and white to blue (F) indicate low to high enrichment level [−log_10_(*P*-value)] for each GO-term or pathway

Skin phenotypes are usually apparent as the initial signs of HGPS within the first year of life (Rork et al., 2014). Dermatologic examination showed HGPS monkeys had sclerodermatous skin that is thin and dry with stippled pigmentation and increased vascular markings on the skull and eyelids (Figs. 1D and 3A). A decrease in epidermal proliferation of HGPS skin was also observed (Fig. 4A), which is in agreement with that of aged human skin (Giangreco et al., 2008). Consistently, fibroblasts from HGPS monkeys had a compromised proliferative ability, increased SA-β-gal staining (Senescence Associated β-galactosidase staining), and heterochromatin loss in culture (Fig. 4B–E). To explore the transcriptomic changes during an early stage of HGPS progression, we isolated skin samples from five-month-old HGPS monkeys and their WT counterparts and sequenced the RNA. Comparisons between WT and HGPS monkeys revealed 201 genes that were upregulated and 248 that were downregulated in HGPS skin (Fig. 5A). Subsequent GO analysis showed that the most significant biological pathways enriched by the upregulated genes were “cytokine and cytokine receptor interaction” and “regulation of inflammation response”, while for the downregulated genes, the most significantly enriched pathway was “keratinization” (Fig. 5B and 5C). We also investigated the genome-wide gene expression changes of HGPS monkeys across multiple-tissues, which has not been reported in any HGPS patient (Gordon Leslie et al., 2016; Fleischer et al., 2018). RNA sequencing identified a total of thousands of differentially expressed genes (DEGs) in HGPS monkey tissue, compared to age-matched wild type controls, with more DEGs in the skin and lungs (Figs. 5D and SF6; Tables S5 and S6). It was also found that progerin led to an enrichment of “chronic inflammation” across tissues, which is consistent with the GO enrichment analysis of the dataset from the skin samples (Fig. 5E, 5F; Tables S5 and S6). These results highlight systemic inflammation as an early and likely critical response to progerin accumulation during the initial stage of HGPS progression and imply that early anti-inflammatory treatment may help mitigate some of the symptoms in HGPS patients.

## Conclusions

This study demonstrates that BE4max enables efficient and precise base editing in the cynomolgus monkey and that the creation of HGPS monkey models can be used for future biomedical research. The HGPS monkeys expressed the mutant progerin protein and exhibited the typical HGPS phenotype. Due to the relatively long reproductive cycle and life span of cynomolgus monkeys, this model may represent a more valuable experimental model than the mouse (Osorio et al., 2011), rabbit (Liu et al., 2018b) or pig (Dorado et al., 2019), in terms of assessing HGPS pathogenesis and the efficacy of related therapeutic intervention.

## Material and Methods

### Animals

Healthy female cynomolgus monkeys (*Macaca fascicularis*), ranging in age from five to eight years with body weights of four to six kg, were selected for use in this study. All animals were housed at the Yunnan Key Laboratory of Primate Biomedical Research (LPBR). All animal procedures were performed following the association for Assessment and Accreditation of Laboratory Animal Care International (AAALAC) for the ethical treatment of primates.

### Preparation of mRNA and sgRNA

PCMV_BE4max_P2A_GFP plasmids were obtained from Addgene (#112099). The plasmid was linearized with the restriction enzyme PmeI, and mRNA was synthesized and purified using an *In Vitro* RNA Transcription Kit (mMESSAGE mMACHINE T7 Ultra kit, Ambion). SgRNA oligos were amplified and transcribed *in vitro* using the GeneArt Precision gRNA Synthesis Kit (Thermo) and purified with the MEGAclear Kit (Thermo) according to the manufacturer’s instructions.

### Oocyte collection and *in vitro* fertilization

Oocyte collection and fertilization were performed as previously described (Niu et al., 2010). In brief, 10 healthy female cynomolgus monkeys aged 5–8 years with regular menstrual cycles were selected as oocyte donors for superovulation, which was performed by intramuscular injection with rhFSH (recombinant human follitropin alpha, GONAL-F, Merck Serono) for 8 days, then rhCG (recombinant human chorionic gonadotropin alpha, OVIDREL, Merck Serono) on day 9 Oocytes were collected by laparoscopic follicular aspiration 32–35 h after rhCG administration. Follicular contents were placed in Hepes-buffered Tyrode’s albumin lactate pyruvate (TALP) medium containing 0.3% BSA at 37 °C. Oocytes were stripped of cumulus cells by pipetting after a brief exposure (<1 min) to hyaluronidase (0.5 mg/mL) in TALP-Hepes to allow visual selection of nuclear maturity metaphase II (MII; first polar body present) oocytes. The maturity oocytes were subjected to intracytoplasmic sperm injection (ICSI) immediately and then cultured in CMRL-1066 containing 10% fetal bovine serum (FBS) at 37 °C in 5% CO_2_. Fertilization was confirmed by the presence of the second polar body and two pronuclei.

### BE4max injection, embryo culture, and transplantation

Six to eight hours after ICSI, the zygotes were injected with a mixture of BE4max mRNA (100 ng/μL) and sgRNA (50 ng/μL) with total volume 5 pL for each zygote. Microinjections were performed in the cytoplasm of oocytes using a microinjection system under standard conditions. Zygotes were then cultured in the chemically defined hamster embryo culture medium-9 (HECM-9) containing 10% fetal bovine serum (FBS, GIBCO) at 37 °C in 5% CaO_2_ to allow embryo development. The culture medium was replaced every other day until the blastocyst stage. The cleaved embryos with high quality at the two-cell to blastocyst stage were transferred into the oviduct of the matched recipient monkeys. Eleven monkeys were used as surrogate recipients. The earliest pregnancy diagnosis was performed by ultrasonography about 20–30 days after the embryo transfer. Both clinical pregnancy and the number of fetuses were confirmed by fetal cardiac activity and presence of a yolk sac as detected by ultrasonography.

### Genomic DNA extraction and sequencing

The genomic DNA from total blood cells and tissues of newborns was extracted by Wizard Genomic DNA Purification Kit (Promega #A1125) according to the manufacturer’s instructions. Sanger sequencing after PCR was performed with primers as follows: F: 5′-ATGTCTTCCCTCCCCTCCTC-3′; R: 5′-ATCTCTCACACTCCAGCCCT-3′.

### Behavioral analysis

Behavioral recording and analysis were performed by three independent trained technicians blinded to the genotypes of the monkeys. The behavior of monkeys in the observation cages was video record without interruption for 30 min between 10:00 a.m. to 11:00 a.m. each day for five days. The observation cage for video recording was the same as the incubator (40 × 85 × 36 cm, W × L × H) for feeding monkeys. For local motion tracking, the trajectory was extracted from the video recorded by a Kinect 2.0 camera (Microsoft, CA). For action analysis, the actions of monkey movement were analyzed by PrimateScan software Version 1.00 (Clever Sys Inc, VA). The PrimateScan can detect monkey behaviors including arousal, awaken, bounce, circle, climb, come down, crouch, drink, eat, hang, jump, land, move, pace, pause, prostrate, remain, rock, scratch, shake the cage, sitting, sleep, somersault, stand, swing, turn, twitch, and urinate. In this analysis, the actions of sit, pause, and sleep were defined as non-activity behavior, and the others were activity behaviors. The duration of activity and non-activity were statistically compared between wild type and HGPS monkeys.

### Hematology

Human growth hormone was detected in machine cobas e-411 (Roche) by hGH Elecsys and cobas -e analyzers kit. The routine blood and biochemical analyses were performed using a BC-2800Vet (Mindray) and VetTest 8008 (BTAP).

### Fibroblast isolation and cell culture

The primary fibroblasts of ear skin were obtained according to previously established methods (Zhang et al., 2018). Briefly, the skin samples were sterilized with 75% ethyl alcohol and washed with PBS, then cut into pieces and adhered to the culture dish after removing the hair and fat tissues. The fibroblasts can be outgrown in about one week. All cells were cultured in DMEM high glucose (Hyclone) medium containing 10% FBS (Gibco), 1% glutamax (Gibco), 1% penicillin/streptomycin (Gibco), 2.5 μg/mL plasmocin (Invitrogen) and 1 mol/L tenofovir under 37 °C, 5% CO_2_ conditions.

### Clonal expansion assay

Fibroblasts at 9th passage were seeded in a 12-well plate (Corning) at a density of 3 × 10^3^ cells per well and cultured for approximately 10 days. Then cells were fixed with 4% formaldehyde for 10 min and stained with crystal violet for 15 min. The cellular colonies were photographed and calculated by ImageJ software.

### Western blot

Western blot was performed according to previous work (Zhang et al., 2018). For tissue protein analysis, frozen samples were ground to a fine powder in liquid nitrogen and lysed with 2× SDS lysis buffer, and for cultured cell protein analysis, cells were harvested and washed with cold PBS and lysed with 2× SDS lysis buffer. Total lysates were quantified using the NANOGROP ONEc (Thermo scientific), approximately 40 μg proteins per lane were separated by SDS-PAGE gel and transferred to polyvinylidene difluoride membranes (Millipore). The membrane was blocked with 5% non-fat milk and probed with the indicated primary antibodies overnight at 37 °C. Antibodies for Western blot were anti-progerin (sc-81611, 1:1,000, Santa Cruz Biotechnology); anti-Lamin A/C (sc-7293, 1:1,000, Santa Cruz Biotechnology), and anti-β-actin (sc-69879, Santa Cruz Biotechnology). After incubating with the horseradish peroxidase-linked secondary antibodies, the signal was detected with an ECL kit (Thermo Fisher). β-actin was used as the loading control.

### qPCR

Total RNA was extracted with TRIzol reagent (Invitrogen) and quantified by NANOGROP ONEc (Thermo scientific). 2 μg of total RNA was reverse transcribed to complementary DNA using the Reverse Transcription Master Mix (Promega). qPCR was carried out with iTaq Universal SYBR Green Supermix (Bio-Rad). The expression level of indicated genes was normalized to GAPDH. The primers used for qPCR: GAPDH-F, 5′- TCGGAGTCAACGGATTTGGT-3′; GAPDH-R, 5′-TTGCCATGGGTGGAATCATA-3′; Progerin-F, 5′- ACTGCACCAGCTCGGGG-3′; Progerin-R, 5′-TCTGGGGGCTCTGGGC-3′.

### X-ray detection

X-ray autoradiography pictures of whole-body skeletons and bones of interest were taken with a digital camera attached (Ralco, SPAIN) on X-ray film (SEDECAL, SPAIN). Body fat detection was measured using hologic discovery wi (HOLOGIC, USA).

### Histology staining

Fresh tissues were fixed with 4% PFA and dehydrated using a gradient alcohol soak, xylene, and then finally embedded in paraffin. Embedded tissues were sliced into sections with a thickness of 5 μm for hematoxylin and eosin staining, according to standard protocols. Fibrosis was examined by Masson staining according to the protocol previously described (Debacq-Chainiaux et al., 2009).

### SA-β-Gal staining

The senescence-associated β-galactosidase assay was performed according to a universally accepted method (Ding et al., 2015; Zhang et al., 2019b).

### Immunofluorescence assay

For cell immunofluorescence staining, about 10^5^ cells were seeded into 24-well plates containing coverslips. After one day of adherent culturing, the cells were fixed with 4% paraformaldehyde for ten minutes, then permeabilized with 0.1% Triton X-100 in PBS and blocked with donkey serum for one hour. For the tissue immunofluorescent staining, tissues were sliced and washed with PBS and blocked with donkey serum in PBS for one hour at room temperature. Subsequently, the cells/tissues were incubated with primary antibodies overnight at 4 °C, washed with PBS three times, followed by incubation with Alexa Fluor 488 (goat anti-rabbit) and Alexa Fluor 633 (goat anti-mouse) conjugated secondary antibodies for one hour. The nuclei were stained with Hoechst 33324 (Invitrogen). TrueVIEW Autofluorescence Quenching Kit (Vector, SP-8400) was applied to slices to reduce tissue autofluorescence. Finally, the coverslips or the tissue slices were mounted with antifade mounting medium (Vectashield) and photographed under a laser scanning confocal microscope (Leica SP5).

### Whole-genome sequencing and bioinformatics analyses of copy-number variations, repeated sequences, and single nucleotide variants

The genomic DNA from 17 monkeys and five untreated blastocyst embryos were used in the WGS analysis. The 15 monkeys included the newborn monkeys and their parents (HGPS #1, BE #2, HGPS #3, HGPS #4, HGPS #5, HGPS #6, WT #1, F #1, F#2, F #3, M #1, M #2, M#3, M #4, and M #5). Five untreated blastocyst embryos (BC #1, BC #2, BC #3, BC #4, and BC #5) and their corresponding parents (F #C and M #C) were used as a control in the analysis of the genome-wide *de novo* mutations (DMNs). The genomic DNA of all samples was extracted by Wizard Genomic DNA Purification Kit (Promega #A1125) according to the manufacturer’s instructions. WGS was performed at mean coverages of 30× by Illumina HiSeq X Ten. The raw data were filtered and trimmed using fastp software (v0.20.0) with the base quality value ≥ 25 (-q 25) (Chen et al., 2018). The qualified short reads were mapped to the reference genome (Macaca_fascicularis_5.0.91_release91 from the ensemble) using BWA (v0.7.17) MEM algorithm (Li and Durbin, 2009). After the initial alignment, Samtools (v1.9) was used to filter multiple mapping reads (mapping quality < 30) and sort aligned BAM files (Li et al., 2009). After Q30 filtering, sambamba markdup was run (v0.7) to remove duplicate reads in the mapped BAM files (Tarasov et al., 2015). The uniquely mapped reads were retained for the copy-number variation (CNV), repeat-sequence analysis and the single-nucleotide variants (SNVs) analysis.

In the CNV analysis, chromosomal sequences were placed into bins of 500 kb in length. The normalized coverage depth for each bin was calculated by dividing the raw coverage depth by the average sequencing depth. The repeat regions annotated for *Macaca fascicularis* by RepeatMasker (db20140131) (http://www.repeatmasker.org) were removed from the genomic sequences before coverage was calculated. The CNV scatterplot was generated using ggplot2.

For the repeat-sequence analysis, Repbase (v.21.11) annotated repeat sequences were used to construct the reference sequence index. Reads were mapped to the indexed repeat sequences, and the mapped reads were grouped into long or short interspersed elements (LINEs or SINEs, respectively), long terminal repeats (LTRs), ribosomal RNAs (rRNAs), and other types of repeats. The number of reads mapped to each type of repeat was normalized concerning the total sequencing depth.

The pipeline for variant analysis was shown in the Fig. S2A. The SNVs and Indels were called out from de-duplicated bam files using Strelka (v2.9.0) (Kim et al., 2018). Then the raw variants were filtered using the following thresholds: “QUAL > 30,” “MQ > 30,” “FILTER==‘PASS,’” “GQ > 30,” and “DP > 20.” Heterozygosity distribution of each filtered SNV was shown in Fig. S3C, which was calculated as the depth of the enriched second base divided by the reference base depth. To identify DMNs with higher confidence, we used TrioDeNovo software (v.0.06) (Wei et al., 2015) to remove background variants in the offspring samples with their corresponding parents as control, following the author’s recommended setting and filter. After obtaining the results from running TrioDeNovo (Raw DNMs), only DMNs with the allele balance between 0.3 and 0.7 that were not shared among offspring (Final DMNs) remained. The number of variants and DMNs after each of the filters were shown in the Fig. S2B. Possible off-target sites with up to five mismatched sites were identified using Cas-OFFinder (http://www.rgenome.net/cas-offinder/). 11,483 possible off-target sequences were identified.

### RNA sequencing data processing

The processing pipeline for RNA-seq data has been reported previously. Pair-end reads were trimmed using Trim Galore (https://github.com/FelixKrueger/TrimGalore). Cleaned reads were mapped to UCSC *Macaca fascicularis* (version macFas5) genome using hisat2 (Kim et al., 2015). Trimmed reads with a mapping quality exceeding 20 were counted by HTSeq (version 0.11.0) (Anders et al., 2015). Differentially expressed genes (DEGs) were revealed using DESeq2 R package (version 1.22.2) using a Benjamini-Hochberg adjusted *P* value (adjusted-*P* value) of less than 0.05 and absolute Log_2_(fold change) of more than 1 (Love et al., 2014). A subsequent GO enrichment analysis was conducted using Metascape (Zhou et al., 2019).

### Data availability

The raw sequence data reported in this paper have been deposited in the Genome Sequence Archive (Wang et al., 2017; National Genomics Data Center and Partners, 2020) in BIG Data Center (Nucleic Acids Res 2018), Beijing Institute of Genomics (BIG), Chinese Academy of Sciences, under accession number CRA002684 that are publicly accessible at https://bigd.big.ac.cn/gsa.

## Electronic supplementary material

Below is the link to the electronic supplementary material.

Supplementary material 1 (PDF 3147 kb)

Supplementary material 2 (XLSX 1913 kb)

Supplementary material 3 (XLSX 1701 kb)
