# Supplementary material for: Generation of a Hutchinson–Gilford progeria syndrome monkey model by base editing
Source: Protein Cell. 2020 Jul 29;11(11):809–24. doi: 10.1007/s13238-020-00740-8 (PMC7647984; doi:10.1007/s13238-020-00740-8)
Supplement: Supplementary file 1 — Supplementary material 1 (PDF 3147 kb) [file 13238_2020_740_MOESM1_ESM.pdf]

Supplementary figure 1. Generation HGPS monkeys.

(A) Summary of embryo microinjection of BE4max and sgRNA.

(B) Photographs of BE #2 and HGPS #3 when they were 3-months old. Scale bar, 0.83 cm.

(C) Sequencing of the sgRNA-targeted regions in the *LMNA* gene of peripheral blood from the indicated monkeys.

(D-E) The on-target editing frequencies at eight cytosine sites within the sgRNA window were analyzed by the WGS data (D, blood; E, umbilical cord).

(F-H) Analyzing the on-target editing frequencies at eight cytosine sites within the sgRNA window by the WGS data of each tissue from the HGPS #4 **(F)**, #5 **(G)**, #6 (**H**) monkeys and corresponding WT monkeys.

A

Oocytes	Injected embryos	Embryos for ET	Pregnancies/ surrogates	Miscarried fetuses	Full-term fetuses	
					Live	Positive (%)
86	68	41	54.55% (6/11)	1	5	4 (80%)

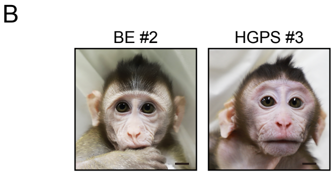

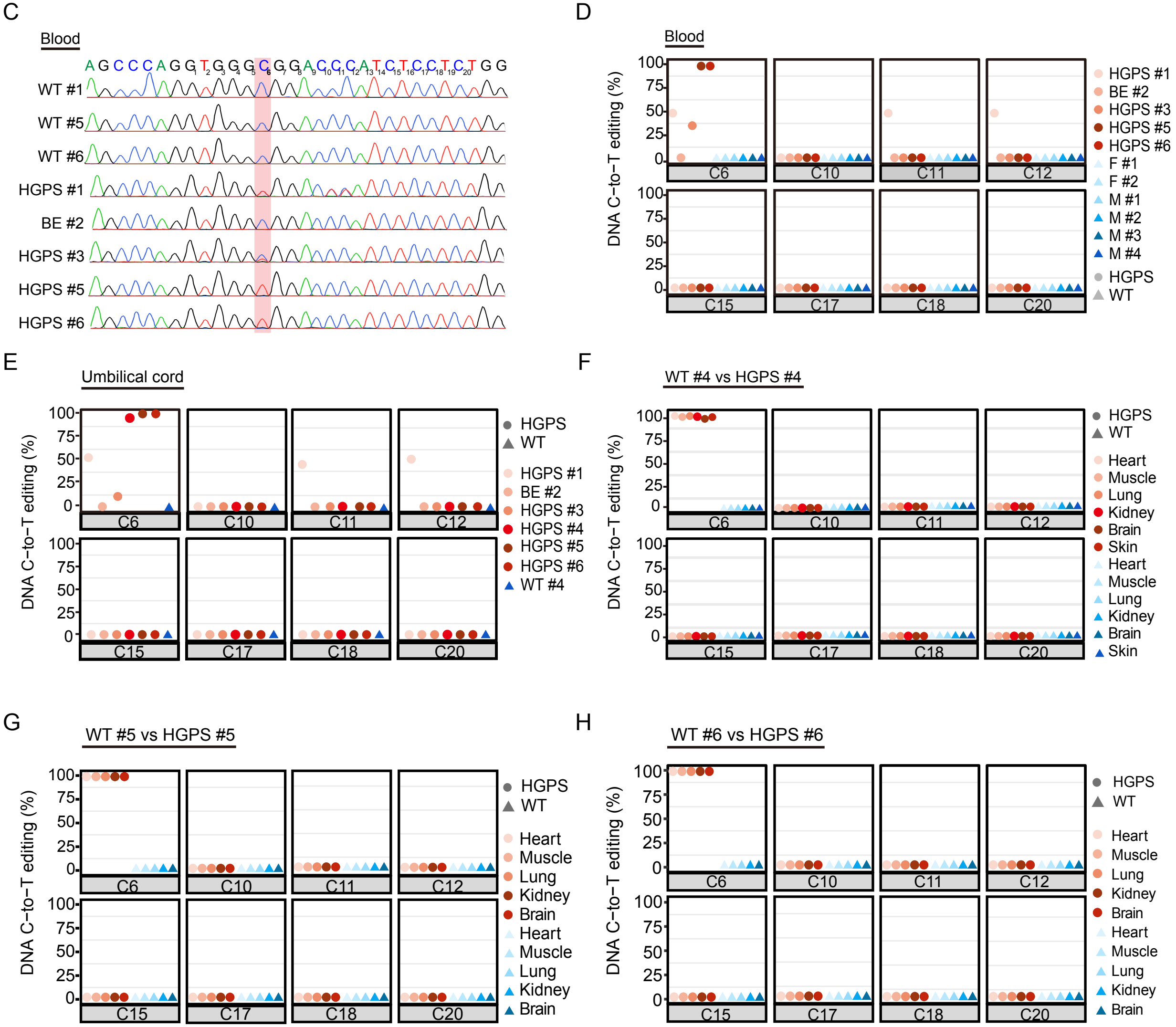

Supplementary figure 2. *LMNA*^{G608G} did not cause chromosomal instability in various tissues in monkeys.

(A) Whole-genomic sequencing of copy number variations (CNV) in various tissues from WT monkeys and HGPS monkeys. Iridescent point, a 500 kb genomic region of each chromosome.

(B) The distribution of different types of repetitive sequences in the genome of various tissues from the WT monkeys and HGPS monkeys. Each type of repeat is marked in the same color: LINE-1, Long Interspersed Element type 1; SINEs, Short Interspersed Elements; LTRs, Long Terminal Repeat (including ERV1, ERV2, EVR3 and other types of LTR); rRNAs and other types of repeat (such as satellite, small nuclear RNA and 'other', as annotated in Repbase). The distributions of repeat reads were not significantly different in the WT and HGPS monkeys, according to the two-sided Wilcoxon signed-rank paired test.

(C) Heterozygosity distribution of SNVs called from Strelka using various tissues from WT monkeys and HGPS monkeys.

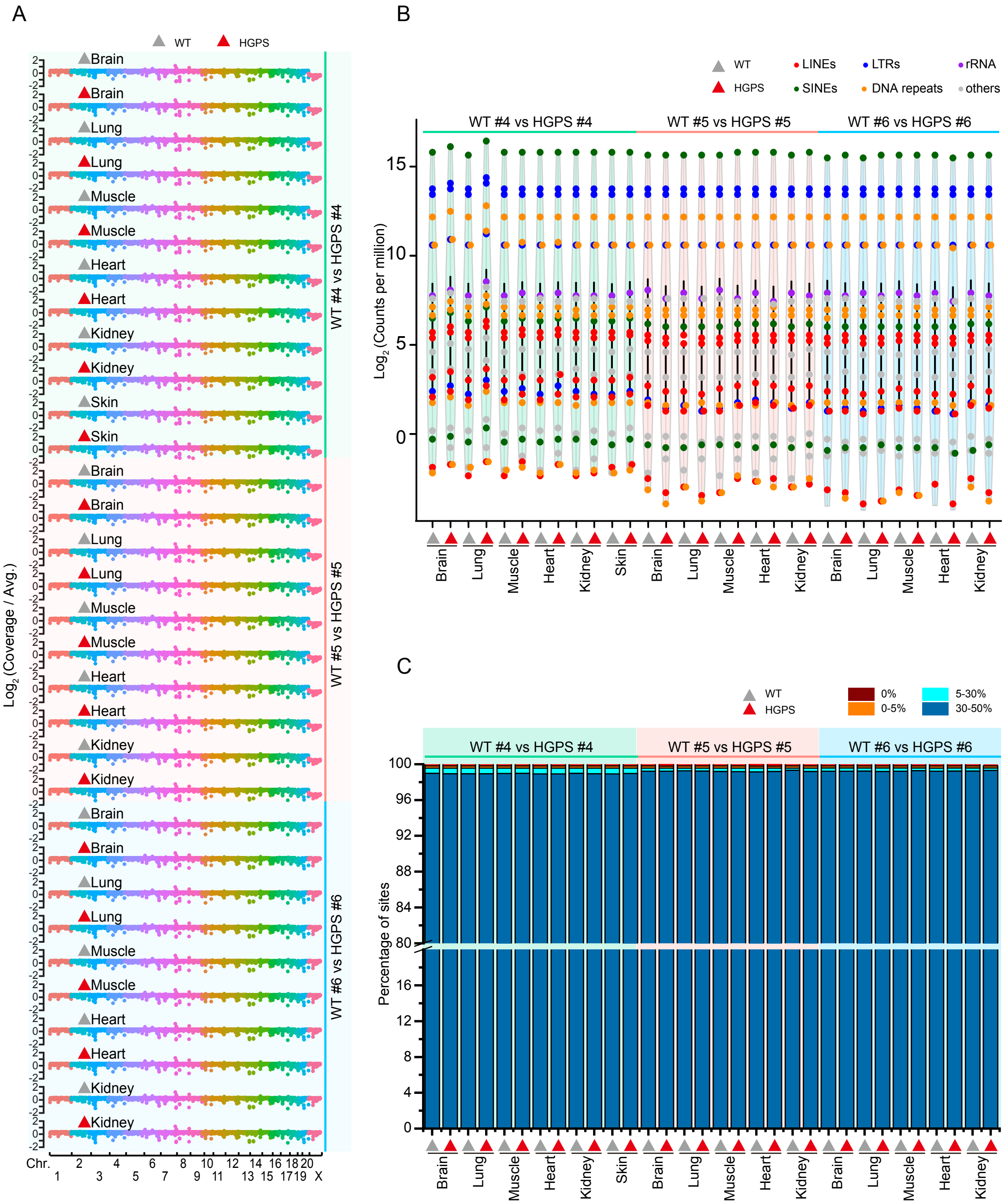
A
WT
HGPS
Brain
Lung
Muscle
Heart
Kidney
Skin
WT #4 vs HGPS #4
WT #5 vs HGPS #5
WT #6 vs HGPS #6
Log2(Coverage / Avg.)
Chr.
1 2 3 4 5 6 7 8 9 10 11 12 13 14 15 16 17 18 19 20 X
B
WT
HGPS
LINEs
SINEs
LTRs
DNA repeats
rRNA
others
Log2(Counts per million)
15
10
5
0
C
0%
0-5%
5-30%
30-50%
Percentage of sites
100
96
92
88
84
80
16
12
8
4
0

Supplementary figure 3. *De novo* SNVs and indels analysis in monkeys.

(A) The pipeline for bioinformatics analysis described in the section of Material and Methods.

(B) Statistics of SNVs and indels in WT and HGPS samples.

(C) Comparing the total number of *de novo* SNVs between WT control samples (WT #1, BC #1, BC #2, BC #3, BC #4, and BC #5) and HGPS monkeys (HGPS #1, BE #2, HGPS #3, HGPS #5, HGPS #6) (*t*-test).

(D) Comparing the distribution of indicated mutations in different genomic regions between WT control samples and HGPS monkeys. The sample information is the same as **(C)** (*t*-test).

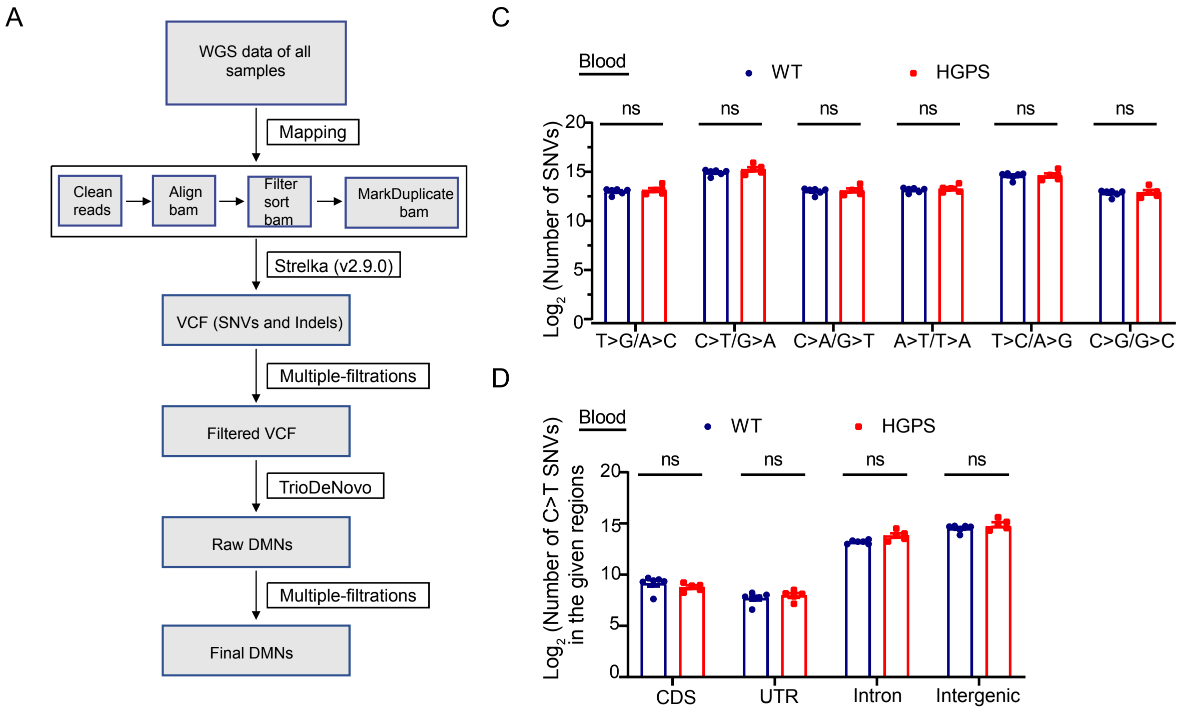

B

	ID	SNVs				Indels			
		Raw variant calls from Stralka	Filtered variants	Raw DMNs TrioDeNovo	Final DMNs	Raw variant calls from Strelka	Filtered variants	Raw DMNS TrioDeNovo	Final DMNs
**WT**	BC #1	16506673	14178834	156880	106793	232424	183051	70054	7199
	BC #2	16628580	13776475	134035	88696	218632	166243	59257	6264
	BC #3	16513285	14176610	162986	111359	234583	188628	74777	7721
	BC #4	16493749	14323958	165406	114335	233156	184799	72011	3160
	BC #5	16540639	14422563	154860	106695	232804	187347	73857	7339
	WT #1	16864057	14594778	77751	66337	236785	184262	73297	6061
**HGPS**	HGPS #1	16757527	14606401	137099	119249	243499	195259	100479	7900
	HGPS #2	16736457	14439883	99593	86556	229919	174022	76339	5932
	HGPS #3	16673329	14919677	203574	173853	263657	223263	169024	13782
	HGPS #5	16815272	14898835	136296	112343	252639	209592	113355	5356
	HGPS #6	16570194	14844965	95389	78059	264353	224189	110120	14303

Supplementary figure 4. Expression of progerin in various tissues of HGPS monkeys.

(A) Quantitative analysis of progerin mRNA expression in various tissues of WT #4 and HGPS #4 monkeys by qPCR. The data from the HGPS monkeys were normalized to the corresponding data obtained from the WT monkeys. Data are mean ± SD. n = 4 wells per condition, ****$P < 0.0001$ (two-way ANOVA).

(B-C) Western blots showed the expression of progerin in the intestine and kidney of HGPS monkeys. For uncropped gels, refer to Source Data.

(D) Western blot showed the absence of progerin in the brain of HGPS monkeys. For uncropped gels, refer to Source Data.

(E-F) Immunofluorescence staining showed the expression of progerin in the small intestine (**E**), and large intestine **(F)** of HGPS monkeys. Scale bar, 25 μm, (zoom: 10 μm). Data are mean ± SD. n = 3 monkeys (WT #4, #5, #6 versus HGPS #4, #5, #6). ***$P < 0.001$ (*t*-test).

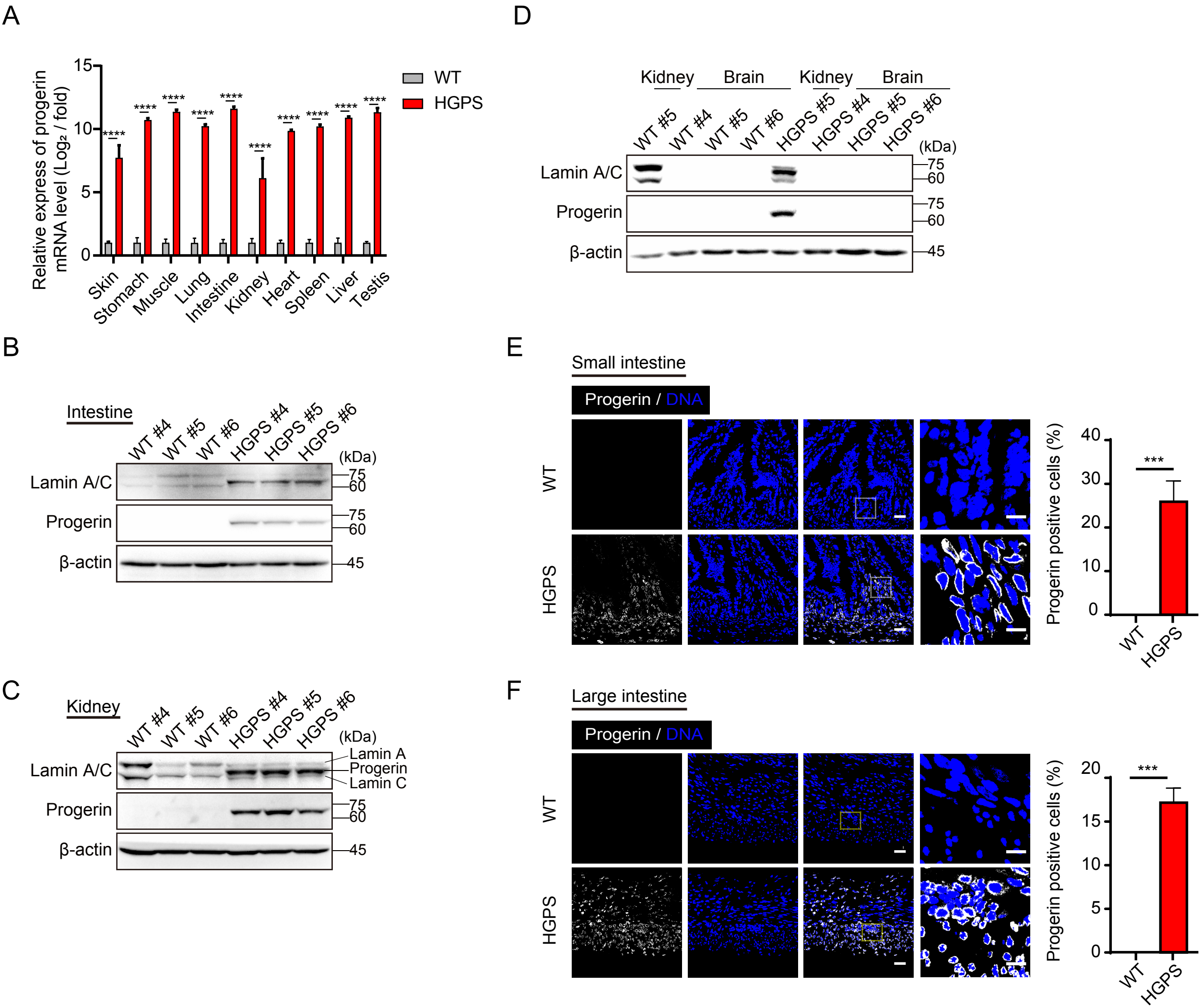
A
Relative express of progerin mRNA level (Log₂ / fold)
15
10
5
0
****
WT
HGPS
Skin
Stomach
Muscle
Lung
Intestine
Kidney
Heart
Spleen
Liver
Testis
B
Intestine
WT #4
WT #5
WT #6
HGPS #4
HGPS #5
HGPS #6
(kDa)
Lamin A/C
Progerin
β-actin
75
60
45
C
Kidney
WT #4
WT #5
WT #6
HGPS #4
HGPS #5
HGPS #6
(kDa)
Lamin A/C
Progerin
β-actin
Lamin A
Progerin
Lamin C
75
60
45
D
Kidney
Brain
Kidney
Brain
WT #5
WT #4
WT #5
WT #6
HGPS #5
HGPS #4
HGPS #5
HGPS #6
(kDa)
Lamin A/C
Progerin
β-actin
75
60
45
E
Small intestine
Progerin / DNA
WT
HGPS
Progerin positive cells (%)
40
30
20
10
0
***
WT
HGPS
F
Large intestine
Progerin / DNA
WT
HGPS
Progerin positive cells (%)
20
15
10
5
0
***
WT
HGPS

Supplementary figure 5. Characterization of HGPS monkeys.

(A) Head circumference of WT and HGPS monkeys after birth. Data was displayed as mean ± SD, n = 3 (WT #1, #5, #6 versus HGPS #1, #5, #6), $^{ns}P > 0.5$ (two-way ANOVA)

(B) Representative photographs showing the appearance of toe malformation in HGPS monkey at 87 days of age. Scale bar, 0.32 cm.

(C) A reduction of activity in HGPS monkeys. Data was presented as mean ± SD, n = 3 (WT #1, #5, #6 versus HGPS #1, #5, #6), $P = 0.0608$ (*t*-test).

(D) An increase of non-activity in HGPS monkeys. Data was presented as mean ± SD, n = 3 (WT #1, #5, #6 versus HGPS #1, #5, #6), $P = 0.1112$ (*t*-test).

(E) There was no difference in blood glucose and HGH (human growth hormone) between WT monkeys and HGPS monkeys. Data was displayed as mean ± SD, n = 3 (WT #1, #5, #6 versus HGPS #1, #5, #6), $^{ns}P > 0.05$ (two-way ANOVA).

(F) Summary of phenotypes observed in monkeys. The number of "+" indicated the different level of phenotype manifestation. HGPS #3 didn't exhibit any typical phenotype of HGPS, possibly due to a low mutation frequency of 11~35%. (N.A. represented not appear).

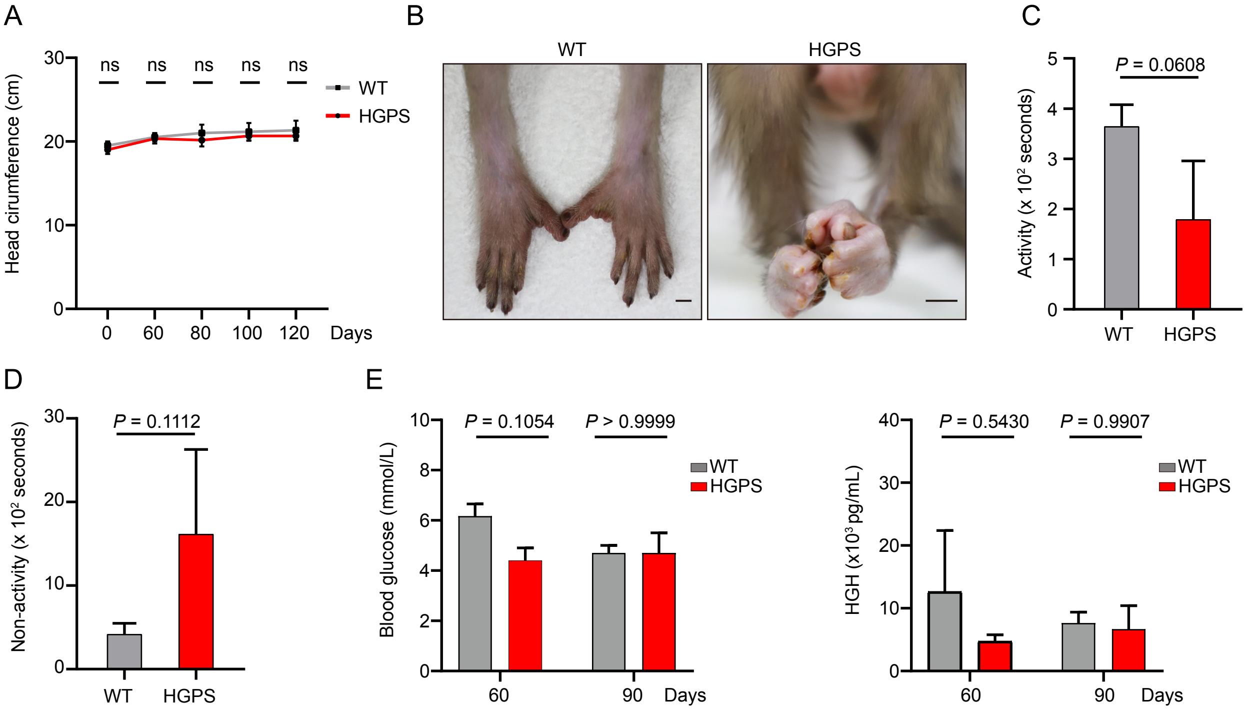

F

Phenotype	HGPS #1	BE #2	HGPS #3	HGPS #4	HGPS #5	HGPS #6
Sex	Female	Female	Female	Male	Male	Female
Age of presentation (months)	4	4	4	0	4	4
Weight (g)	500	500	950	260	470	410
Height (cm)	17	17	17	14	16	15.5
Body mass index (g/cm^2)	1.73	1.73	12.38	1.33	1.84	1.71
Alopecia	+	-	-	N.A.	++	++
Scleroderma-like skin atrophy	+	-	-	N.A.	+	++
Decrustation	++	-	-	N.A.	+	+++
Mottled pigmentation of the skin	+	-	-	N.A.	++	++
Eyes proptosis	+	-	-	N.A.	+	++
Tear ducts sensitive	+	-	-	N.A.	-	++
Nasal discharge	+	-	-	N.A.	+	++
Panting hard	+	-	-	N.A.	+	++
Micrognathia	+	-	-	N.A.	+	+
Disarry teeth	+	-	-	N.A.	+	+
Coracoid mouth	+	-	-	N.A.	+	+
Recurrent diarrhea	+	-	-	N.A.	+	++
Acro-osteolysis	+	-	-	N.A.	++	++
Joint stiffness/arthritis	+	-	-	N.A.	++	++

Supplementary figure 6. Transcriptome features of HGPS monkeys.

(A) Heat map showed the Euclidean distance between WT monkeys and HGPS monkeys in indicated tissues based on the expression levels of all coding genes. The color keys from blue to yellow indicated low to high Euclidean distance. Two experimental repeats are shown.

(B) Heat maps showed upregulated genes (left panel) and downregulated genes (right panel) between WT monkeys and HGPS monkeys in various tissues. The color keys from white to red and blue to white indicated low to high $\log_2$ (fold change) for upregulated and downregulated genes between WT monkeys (WT #5 and #6) and HGPS monkeys (HGPS #5 and #6), respectively.

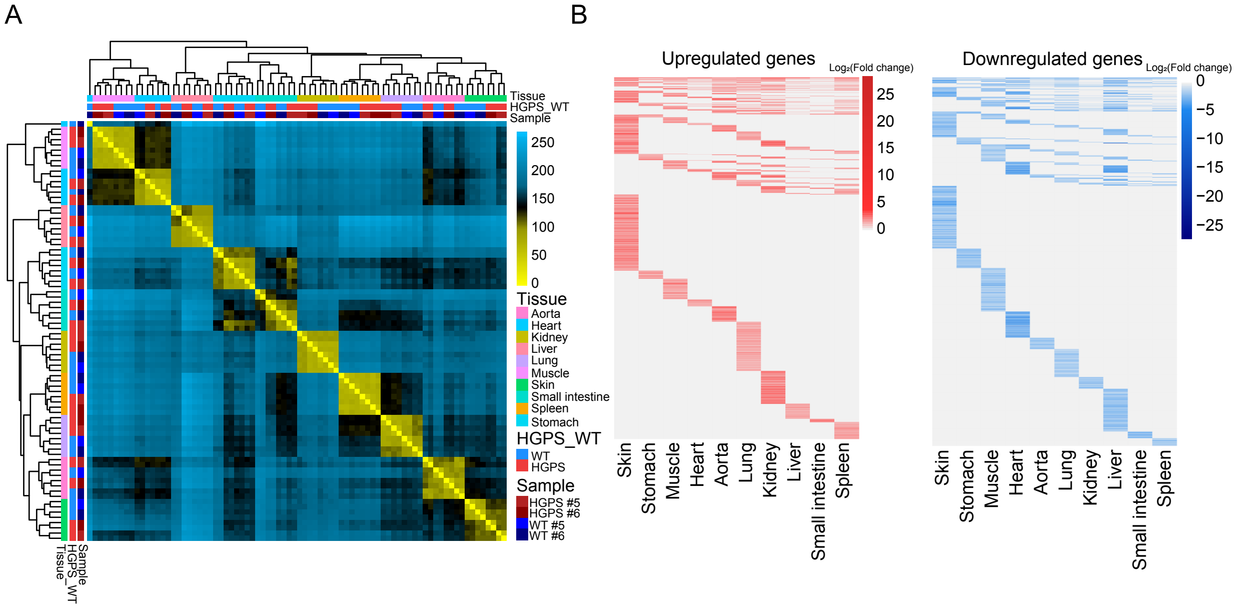
A
Tissue
HGPS_WT
Sample
250
200
150
100
50
0
Tissue
Aorta
Heart
Kidney
Liver
Lung
Muscle
Skin
Small intestine
Spleen
Stomach
HGPS_WT
WT
HGPS
Sample
HGPS #5
HGPS #6
WT #5
WT #6
B
Upregulated genes
Log₂(Fold change)
25
20
15
10
5
0
Skin
Stomach
Muscle
Heart
Aorta
Lung
Kidney
Liver
Small intestine
Spleen
Downregulated genes
Log₂(Fold change)
0
−5
−10
−15
−20
−25
Skin
Stomach
Muscle
Heart
Aorta
Lung
Kidney
Liver
Small intestine
Spleen

Source data. The original photograph involved in the representative figures of WB.

(A-E) The enlarged screenshots of indicated WB results shown in figure 2C **(A)**, figure 2D **(B)**, supplementary figure 4B **(C)**, supplementary figure 4C **(D)**, and supplementary figure 4D **(E)**.

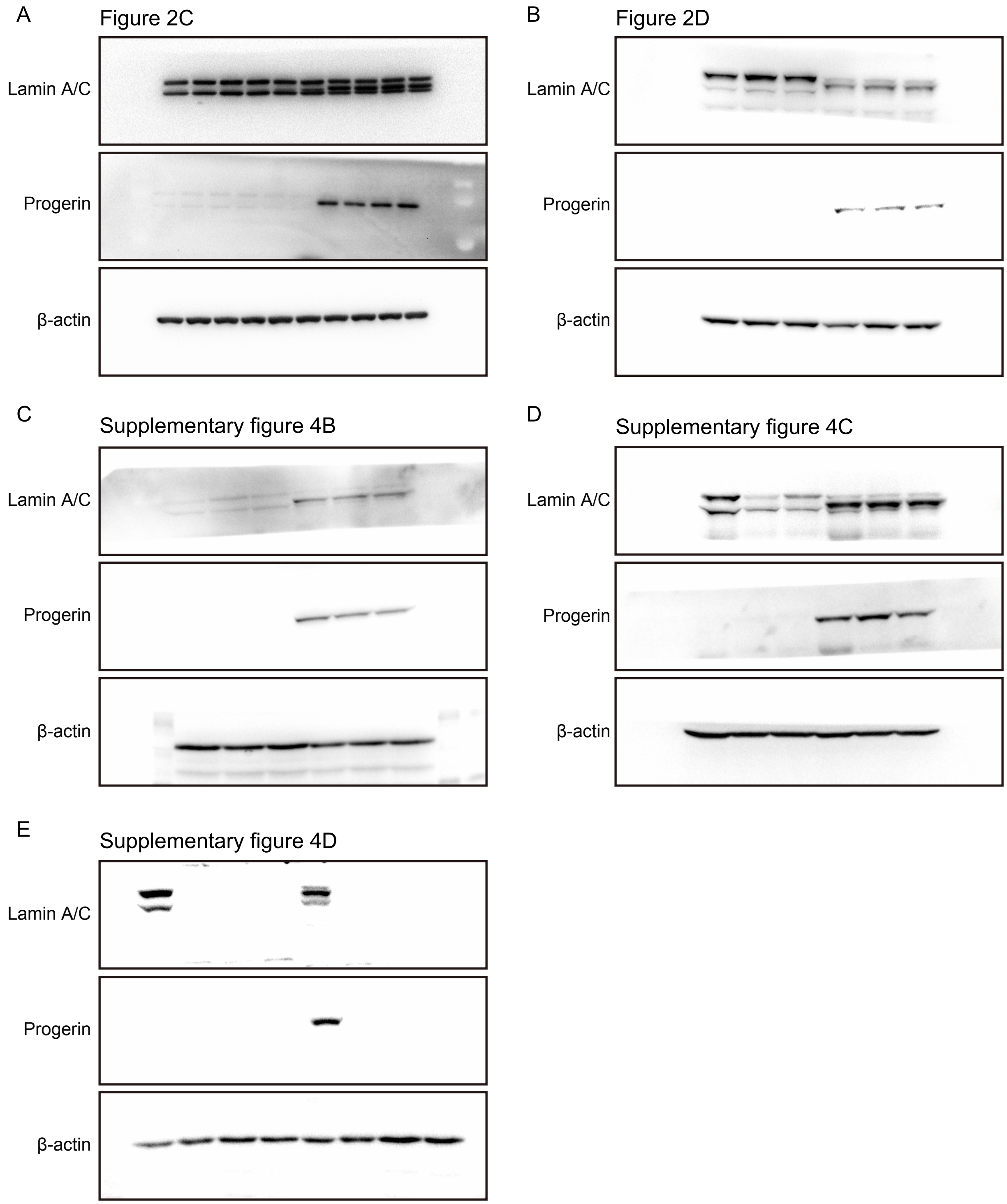

A
Figure 2C
Lamin A/C
Progerin
β-actin
B
Figure 2D
Lamin A/C
Progerin
β-actin
C
Supplementary figure 4B
Lamin A/C
Progerin
β-actin
D
Supplementary figure 4C
Lamin A/C
Progerin
β-actin
E
Supplementary figure 4D
Lamin A/C
Progerin
β-actin

Supplementary Tables

Table S1

ID	Sex	Gestation cycle (days)	Birth weight (kg)	Birth height (cm)	Body mass index (kg/cm^2) (x10^{-3})	Life span (days)
**HGPS #1**	♀	159	0.325	13	1.92	alive
**BE #2**	♀	159	0.4	14.5	1.9	alive
**HGPS #3**	♀	161	0.415	14	2.12	alive
**HGPS #4**	♂	146	0.26	14	1.33	0
**HGPS #5**	♂	166	0.36	14	1.84	151
**HGPS #6**	♀	165	0.43	14	2.19	132
**WT #1**	♀	155	0.36	14	1.84	alive
**WT #4**	♂	145	0.31	14	1.58	0
**WT #5**	♂	155	0.4	14.1	2.01	136
**WT #6**	♀	155	0.36	13.8	1.89	158

Table S1. Summary of monkeys' information. Summary of monkey cohorts used in this study.

Table S2

Sample type	Monkey	G5	C6	G7	G8	A9	C10	C11	C12	A13
**Blood**	WT #1	0	0	0	0	0	0	0	0	0
	HGPS #1	0	0.49	0	0	0	0	0.48	0.46	0
	BE #2	0	0	0	0	0	0	0	0	0
	HGPS #3	0	0.35	0	0	0	0	0	0	0
	HGPS #5	0	1	0	0	0	0	0	0	0
	HGPS #6	0	1	0	0	0	0	0	0	0
	F1	0	0	0	0	0	0	0	0	0
	F2	0	0	0	0	0	0	0	0	0
	F3	0	0	0	0.02	0	0	0	0	0
	M1	0	0	0	0	0	0	0	0	0
	M2	0	0	0	0	0	0	0	0	0
	M3	0	0	0	0	0	0	0	0	0
	M4	0	0	0	0	0	0	0	0	0
	M5	0	0	0	0	0	0	0	0	0
**Umbilical cord**	WT #4	0	0	0	0	0	0	0	0	0
	HGPS #1	0	0.53	0	0	0	0	0.55	0.55	0
	BE #2	0	0	0	0	0	0	0	0	0
	HGPS #3	0	0.11	0	0	0	0	0	0	0
	HGPS #4	0	0.96	0	0	0	0	0	0	0
	HGPS #5	0	1	0	0	0	0	0	0	0
	HGPS #6	0	1	0	0	0	0	0	0	0
**Heart**	WT #4	0	0	0	0	0	0	0	0	0
	HGPS #4	0	0.97	0	0	0	0	0	0	0
	WT #5	0	0	0	0	0	0	0	0	0
	HGPS #5	0	1	0	0	0	0	0	0	0

	WT #6	0	0	0	0	0	0	0	0	0
	HGPS #6	0	1	0	0	0	0	0	0	0
**Muscle**	WT #4	0	0	0	0	0	0	0	0	0
	HGPS #4	0	0.96	0	0	0	0	0	0	0
	WT #5	0	0	0	0	0	0	0	0	0
	HGPS #5	0	1	0	0	0	0	0	0	0
	WT #6	0	0	0	0	0	0	0	0	0
	HGPS #6	0	1	0	0	0	0	0	0	0
**Brain**	WT #4	0	0	0	0	0	0	0	0	0
	HGPS #4	0	0.94	0	0	0	0	0	0	0
	WT #5	0	0	0	0	0	0	0	0	0
	HGPS #5	0	1	0	0	0	0	0	0	0
	WT #6	0	0	0	0	0	0	0	0	0
	HGPS #6	0	1	0	0	0	0	0	0	0
**Lung**	WT #4	0	0	0	0	0	0	0	0	0
	HGPS #4	0	0.97	0	0	0	0	0	0	0
	WT #5	0	0	0	0	0	0	0	0	0
	HGPS #5	0	1	0	0	0	0	0	0	0
	WT #6	0	0	0	0	0	0	0	0	0
	HGPS #6	0	1	0	0	0	0	0	0	0
**Kidney**	WT #4	0	0	0	0	0	0	0	0	0
	HGPS #4	0	0.96	0	0	0	0	0	0	0
	WT #5	0	0	0	0	0	0	0	0	0
	HGPS #5	0	1	0	0	0	0	0	0	0
	WT #6	0	0	0	0	0	0	0	0	0
	HGPS #6	0	1	0	0	0	0	0	0	0

Table S2. Summarized the base editing frequency in seven tissues.

Table S3

<table>
<tr><td rowspan="4">No. of mismatches</td><td colspan="3" rowspan="3">PAM</td><td>CDS</td><td colspan="5" rowspan="3">Off-targets in mutant monkey</td></tr>
<tr><td>UTR</td></tr>
<tr><td>Intron</td></tr>
<tr><td>NGG</td><td>NAG</td><td>NNN</td><td>Intergenic</td><td>HGPS #1</td><td>BE #2</td><td>HGPS #3</td><td>HGPS #5</td><td>HGPS #6</td></tr>
<tr><td rowspan="4">0</td><td rowspan="4">1</td><td rowspan="4">0</td><td rowspan="4">0</td><td>1</td><td>N.A.</td><td>N.A.</td><td>N.A.</td><td>N.A.</td><td>N.A.</td></tr>
<tr><td>0</td><td>N.A.</td><td>N.A.</td><td>N.A.</td><td>N.A.</td><td>N.A.</td></tr>
<tr><td>0</td><td>N.A.</td><td>N.A.</td><td>N.A.</td><td>N.A.</td><td>N.A.</td></tr>
<tr><td>0</td><td>N.A.</td><td>N.A.</td><td>N.A.</td><td>N.A.</td><td>N.A.</td></tr>
<tr><td rowspan="4">1</td><td rowspan="4">0</td><td rowspan="4">0</td><td rowspan="4">0</td><td>0</td><td>0</td><td>0</td><td>0</td><td>0</td><td>0</td></tr>
<tr><td>0</td><td>0</td><td>0</td><td>0</td><td>0</td><td>0</td></tr>
<tr><td>0</td><td>0</td><td>0</td><td>0</td><td>0</td><td>0</td></tr>
<tr><td>0</td><td>0</td><td>0</td><td>0</td><td>0</td><td>0</td></tr>
<tr><td rowspan="4">2</td><td rowspan="4">0</td><td rowspan="4">0</td><td rowspan="4">7</td><td>1</td><td>0</td><td>0</td><td>0</td><td>0</td><td>0</td></tr>
<tr><td>1</td><td>0</td><td>0</td><td>0</td><td>0</td><td>0</td></tr>
<tr><td>1</td><td>0</td><td>0</td><td>0</td><td>0</td><td>0</td></tr>
<tr><td>4</td><td>0</td><td>0</td><td>0</td><td>0</td><td>0</td></tr>
<tr><td rowspan="4">3</td><td rowspan="4">9</td><td rowspan="4">6</td><td rowspan="4">71</td><td>3</td><td>0</td><td>0</td><td>0</td><td>0</td><td>0</td></tr>
<tr><td>5</td><td>0</td><td>0</td><td>0</td><td>0</td><td>0</td></tr>
<tr><td>37</td><td>0</td><td>0</td><td>0</td><td>0</td><td>0</td></tr>
<tr><td>41</td><td>1</td><td>0</td><td>0</td><td>0</td><td>0</td></tr>
<tr><td rowspan="4">4</td><td rowspan="4">70</td><td rowspan="4">70</td><td rowspan="4">926</td><td>52</td><td>0</td><td>0</td><td>0</td><td>0</td><td>0</td></tr>
<tr><td>27</td><td>0</td><td>0</td><td>0</td><td>0</td><td>0</td></tr>
<tr><td>430</td><td>3</td><td>2</td><td>0</td><td>1</td><td>1</td></tr>
<tr><td>557</td><td>0</td><td>0</td><td>2</td><td>2</td><td>2</td></tr>
<tr><td>5</td><td>781</td><td>671</td><td>8871</td><td>495</td><td>0</td><td>0</td><td>0</td><td>0</td><td>0</td></tr>
</table>

				263	0	0	0	0	0
				4065	7	8	8	9	10
				5500	8	7	17	8	9

Table S3. The predicted off-target sites. Whole-genome sequencing data did not reveal notable mutations in the potential off-target sites predicted based on sequences similar to the sgRNA sequence. The presumed PAM sequences and genomic locations of these sites are shown.

Table S4.

	HGPS #1	BE #2	HGPS #3	HGPS #5	HGPS #6	WT #1	WT #2	WT #3
**WBC (10^9/L)**	11.20	12.10	20.40	14.20	16.90	12.60	11.10	25.80
**Lymph (10^9/L)**	5.60	4.00	12.90	6.20	9.20	7.70	8.60	15.60
**Mon (10^9/L)**	1.10	1.30	3.40	0.90	2.60	2.00	0.80	5.30
**Gran (10^9/L)**	4.00	6.80	4.10	7.10	5.10	2.90	1.70	4.90
**Lymph (%)**	50.30	32.70	62.80	44.00	54.50	61.50	77.60	60.40
**Mon (%)**	9.80	11.10	16.90	6.60	15.60	15.80	6.80	20.60
**Gran (%)**	39.90	56.20	20.30	49.40	29.90	22.70	15.60	19.00
**RBC (10^{12}/L)**	6.73	6.98	6.79	5.66	5.94	6.31	6.85	6.41
**HGB (g/dL)**	11.00	15.10	14.90	12.70	13.40	13.80	15.80	14.00
**HCT (%)**	40.70	51.80	51.30	43.20	47.00	47.30	51.20	51.80
**MCV (fL)**	60.60	74.30	75.60	76.50	79.20	75.10	77.70	80.90
**MCH (pg)**	16.30	21.60	21.90	22.40	22.50	21.80	23.00	21.80
**MCHC (g/dL)**	27.00	29.10	29.00	29.30	28.50	29.10	29.60	27.00
**RDW (%)**	14.20	14.40	15.30	14.70	16.20	14.20	15.40	16.30
**PLT (10^9/L)**	812.00	657.00	336.00	466.00	674.00	724.00	554.00	728.00
**MPV (fL)**	8.00	8.10	8.50	8.90	9.50	9.00	8.40	7.80
**PDW**	17.00	17.00	17.00	16.80	16.70	17.00	17.40	16.90
**PCT (%)**	0.65	0.53	0.29	0.41	0.64	0.65	0.47	0.57
**URIC< (μmol/L)**	8.00	6.00	6.00	6.00	6.00	6.00	6.00	6.00
**CREA (μmol/L)**	28.00	48.00	36.00	57.00	64.00	35.00	35.00	56.00
**TBIL (μmol/L)**	2.00	5.00	2.00	7.00	6.00	2.00	2.00	4.00
**CHOL(mmol/L)**	3.41	2.92	3.56	6.87	3.12	4.31	5.82	2.92
**TRIG (mmol/L)**	0.68	0.36	1.02	0.99	1.10	0.42	0.71	0.32
**UREA (mmol/L)**	6.70	3.10	2.40	6.40	4.50	4.40	7.90	6.70
**AST (U/L)**	17.00	11.00	0.00	18.00	14.00	11.00	29.00	57.00
**ALT (U/L)**	26.00	23.00	36.00	36.00	29.00	56.00	77.00	61.00

**GLU (mmol/L)**	7.93	4.26	4.33	1.58	2.62	4.57	3.50	3.81

Table S4. Summary of serum analysis.

Table S5. Summary of upregulated DEGs between WT and HGPS monkeys.

Table S6. Summary of downregulated DEGs between WT and HGPS monkeys.
